# Visuomotor resonance in autism spectrum disorders

**DOI:** 10.3389/fnint.2012.00110

**Published:** 2012-11-22

**Authors:** Cristina Becchio, Umberto Castiello

**Affiliations:** ^1^Dipartimento di Psicologia, Centro di Scienza Cognitiva, Università di TorinoTurin, Italy; ^2^Dipartimento di Psicologia Generale, Università di PadovaPadova, Italy

**Keywords:** autism, visuomotor resonance, motor facilitation, mirror system, social cognition

## Abstract

When we observe the actions performed by others, our motor system “resonates” along with that of the observed agent. Is a similar visuomotor resonant response observed in autism spectrum disorders (ASD)? Studies investigating action observation in ASD have yielded inconsistent findings. In this perspective article we examine behavioral and neuroscientific evidence in favor of visuomotor resonance in ASD, and consider the possible role of action-perception coupling in social cognition. We distinguish between different aspects of visuomotor resonance and conclude that while some aspects may be preserved in ASD, abnormalities exist in the way individuals with ASD convert visual information from observed actions into a program for motor execution. Such abnormalities, we surmise, may contribute to but also depend on the difficulties that individuals with ASD encounter during social interaction.

## Introduction

When we observe the actions performed by others, our motor system “resonates” along with that of the observed agent. The prevalent assumption in the literature is that this motor resonance to others' actions depends on a common coding for action execution and observation: observing the actions of others activates, within the observer's motor system, the same motor programs used to execute the observed actions (see Blakemore and Frith, 2005, for a review). Is a similar visuomotor resonant response observed in autism spectrum disorders (ASD)? In the following, we review evidence in favor of visuomotor resonance in neurotypical and participants with ASD. First, we consider evidence stemming from behavioral and neuroscientific methods. Following this groundwork, we examine some of the factors that, by modulating visuomotor resonance, may help integrating apparently divergent findings. Finally, we consider the possible role of visuomotor resonance in social cognition. We speculate that, in accordance with associative proposals, abnormalities in visuomotor resonance may contribute to, but also depend on the difficulties that individuals with ASD encounter during social interaction.

## Motor facilitation and interference by action observation

### When action observation facilitates action execution

Interactions between action observation and action execution can be tested by looking at compatibility effects during movement execution and observation paradigms. If action execution and observation share a common coding, then observing an action should *facilitate* motor performance of a similar action. In accordance with this hypothesis, in neurotypical participants reaction times to initiate a tapping action have been shown to be faster in response to the observation of a task-irrelevant congruent movement (tapping a finger) than in response to the observation of a task-irrelevant incongruent movement (lifting a finger; e.g., Brass et al., 2000, 2001). Similarly, reaction times to initiate a grasping action have been demonstrated to be faster following the observation of a photograph of the final hand posture necessary for the grasping action relative to an incompatible hand posture (Craighero et al., 2002). These compatibility effects have been replicated for various pairs of actions, with both static action stimuli (stills depicting the end of the movement) and dynamic action stimuli (videos), in choice and simple reaction time tasks (for review, see Heyes, 2011). Using a choice reaction time task, Bird et al. (2007) report that ASD participants show an equivalent compatibility effect: responses on compatible trials (e.g., performing an opening hand movement after observing a hand in an opening position) were faster than those on incompatible trials (e.g., performing an opening hand movement after observing a hand in a closing position). As for typically developing controls, the compatibility effect was greater when responses were made to human than to robotic hand postures. These findings have been interpreted as motor facilitation in terms of faster response initiation when there is high compatibility of topographical features of task-irrelevant action stimuli and the prepared action; however, an equally plausible interpretation is that response initiation is delayed when the topographical features of task-irrelevant action stimuli are incompatible with the movement being prepared (Blakemore and Frith, 2005; Heyes, 2011). Evidence that compatibility effects are due, at least in part, to interference rather than facilitation comes from studies showing that responding is slower in imitatively incompatible trials than in baseline trials where the task-relevant cue is presented in the absence of a task-irrelevant movement stimulus (Brass et al., 2000; Bertenthal et al., 2006; Gillmeister et al., 2008).

An alternative approach to motor facilitation, taken by Castiello et al. (2002) and Edwards et al. (2003), has been to investigate motor priming by observation of prehensile movements. Neurotypical participants observed a grasping action directed to an object (e.g., a small object) and then had to grasp either the same object (small object) or a different object (large object). Results revealed a reliable priming effect on the kinematics of the reach-to-grasp movements. Reaching was faster and grasping was more precise when the observed object was the same size as the object to be reached, suggesting that observation of an action facilitated subsequent execution of a matching action.

**Figure 1:**
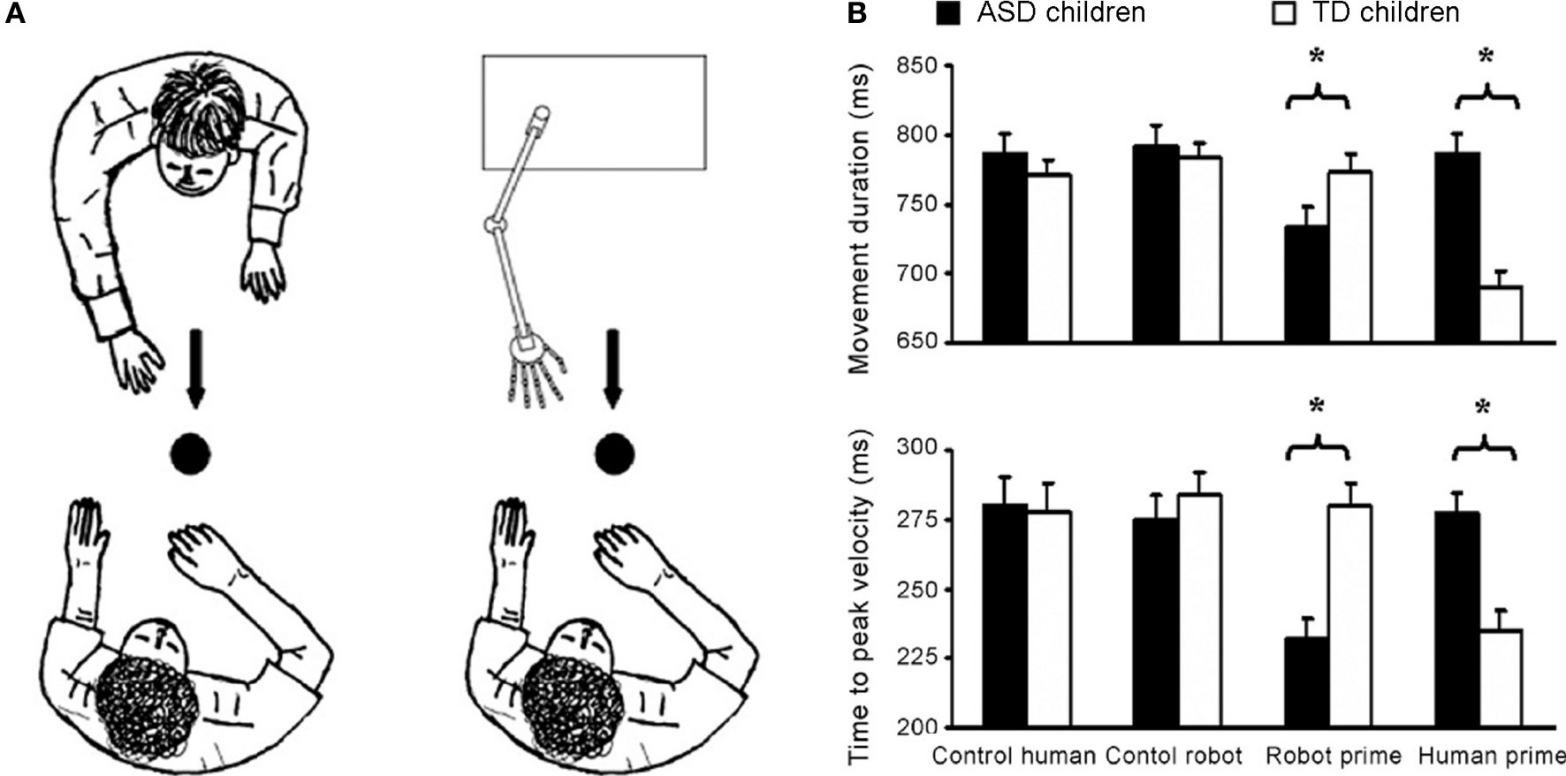
**Motor facilitation for robotic but not for human hand movements. (A)** Experimental set up. Participants were requested to observe either a human or a robotic arm model performing a reach-to-grasp action toward a spherical object. Subsequently, they were asked to perform the same action toward the same object. Two “control” conditions in which participants performed the movement in the presence of either the static human or robot model were also included. **(B)** Graphical representation of the significant interaction between group (autistic children, typically developing children) and condition (control human, control robot, robot prime, and human prime) for movement duration and time to peak velocity. For the normally developing children movement duration was shorter and the time to peak velocity was reached earlier for the “human prime” than for the “robot prime” and the two “control” conditions (*ps* < 0.001). For the children with ASD movement duration was shorter and time to peak velocity was earlier for the “robot prime” than for the “human” and the two “control” conditions (*ps* < 0.001). Bars represent the standard errors of the means. Asterisks indicate significance for the main contrasts of interest (adapted from Pierno et al., 2008).

Using a similar visuomotor priming paradigm, Pierno et al. (2006) found that motor facilitation is impaired in participants with ASD. Whereas typically developing children showed facilitation effects in terms of movement speed following the observation of the model grasping or simply gazing at an object, children with autism did not show any motor facilitation from action or gaze (Pierno et al., 2006). These findings suggest that, in contrast to typically developing children, in children with ASD information from others' gaze and action fails to automatically modulate motor execution. A subsequent study by Pierno et al. (2008) reports motor facilitation for robotic but not for human hand movements: children with autism were facilitated—as revealed by a faster movement duration and an anticipated peak velocity—when primed by a robotic but not by a human arm movement. The opposite pattern was found for typically developing controls (see Figure 1).

### When action observation interferes with action execution

If the motor system is geared up to execute observed movement, this should result in *interference* when the observed movement is qualitatively different from the performed movement. This has been demonstrated for simultaneous movement performance-observation (Kilner et al., 2003; see also, Stanley et al., 2007; Hardwick and Edwards, 2012). Kilner et al. (2003) asked participants to make either horizontal or vertical intransitive and continuous arm movements in time with the movements of an experimenter so that the two peoples' movements were either congruent (i.e., both moving in the same plane) or incongruent (i.e., participant moving their arm in plane perpendicular to that of experimenter). Finger tip movement variability (as measured in the orthogonal plane) was greater in the orthogonal plane for incongruent than for congruent conditions. A similar pattern of interference has been reported during observation of moving dot stimuli when the participants were informed that they were observing prerecorded human movement (Stanley et al., 2007).

Using the same paradigm, Gowen et al. (2008) found an equivalent interference effect in control participants and participants with ASD: both groups displayed greater error plane deviation during incongruent compared to congruent trials. Compared to control participants, however, ASD participants showed a different pattern of movement variability (calculated by summing congruent and incongruent error plane deviation). Whereas control participants made generally more variable movements during observation of biological dot motion stimuli than during observation of arm movements, the reverse was true for ASD participants. These results may indicate reduced visuomotor integration in ASD so that the visual properties of the observed dot motion are less efficiently integrated into the executed movement during continuous movement execution and observation paradigms.

Becchio et al. (2007) found that in comparison with matched, typically developing controls, children with ASD are immune to motor interference in the form of transfer of distractor-mediated effects. In a series of experiments, participants observed a model reaching toward an object presented in isolation or flanked by a distractor object. Immediately after the completion of the model's action, they were asked to perform the same action on the same object, but in absence of the distractor object. Despite the distractor being removed, distractor-mediated effects were evident in the kinematics of typically developing children. Consistent with prior evidence, transfer of interference was also present when the model simply looked at the target in the presence of the distractor object, suggesting that, even in the absence of any overtly executed action, motor intentions read in other's people gaze may cause interference effects (Castiello, 2003). In contrast, children with ASD did not show any interference effect either from action or from gaze observation.

Immunity from the effects of a gaze-based social context is further confirmed by Schilbach et al. (2011) showing that individuals with ASD are not susceptible to the modulatory effect of gaze cues in a stimulus response compatibility paradigm. Participants were asked to generate spatially congruent or incongruent motor responses to changes in a face, a face-like and an object stimulus. Whereas in the comparison group being looked at by a virtual other led to a reduction of reaction time costs associated with generating a spatially incongruent response, this effect was not observed in the ASD group.

## Mirror effects to action observation

At a neural level support for the common coding hypothesis comes from studies showing that action observation recruits the observer's motor system. Evidence for common coding has been found at the level of single neurons, the so-called mirror neurons, in the premotor cortex of macaque monkeys (for review, see Rizzolatti and Sinigaglia, 2010). In humans, the first demonstration of covert motor activation during action observation was provided by Fadiga et al. (1995) using transcranial magnetic stimulation (TMS). TMS was applied to the sector of primary motor cortex (M1) that represents the hand, and motor-evoked potentials (MEPs) were recorded from contralateral hand muscles during the passive observation of hand movements. Observing hand actions determined an enhancement of MEPs in the same muscular groups used in executing those actions (for review, see Fadiga et al., 2005). Corticospinal facilitation during action observation has since been replicated in numerous studies, and it is now well-established that in neurotypical observers the mere observation of others' actions modulates the excitability of the observer's corticospinal circuitry involved in the execution of the same movements (e.g., Strafella and Paus, 2000; Aziz-Zadeh et al., 2002; Maeda et al., 2002; Urgesi et al., 2006; Cavallo et al., 2012). Applying TMS over M1 during observation of intransitive, meaningless finger movements, Théoret et al. (2005) found that overall modulation of M1 excitability during action observation is significantly lower in individuals with ASD compared with matched controls.

**Figure 2:**
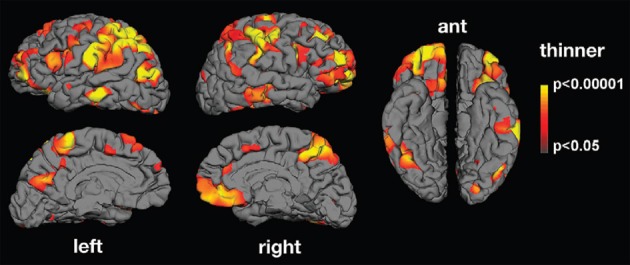
**Mean thickness difference significance maps.** Lateral, medial, and ventral views of the brain showing areas presenting cortical thinning in the autism group compared with neurotypical controls. Significant thinning was found in areas belonging to the MNS (inferior frontal gyrus, inferior parietal lobule, and superior temporal sulcus) as well as in areas involved in facial expression production and recognition (face regions in sensory and motor cortex and in middle temporal gyrus), imitation (superior parietal lobule), and social cognition (prefrontal cortex, anterior cingulate, medial parietal cortex, supramarginal gyrus, and middle and inferior temporal cortex; from Hadjikhani et al., 2006).

Along the same lines, abnormalities in the neural mechanism matching action observation and execution in ASD have been reported using electroencephalography (EEG; Oberman et al., 2005, 2008; Martineau et al., 2008), magnetoencephalography (MEG; Nishitani et al., 2004) and functional magnetic resonance imaging (fMRI; Williams et al., 2006; Martineau et al., 2010). Oberman et al. (2005) found that in comparison to typically developing controls, mu wave suppression—an index of mirror neuron activity—is reduced in ASD during action observation. Nishitani et al. (2004) report abnormalities in the cortical activation chain of ASD participants while they imitate orofacial gestures. MEG responses were normal in strength and timing at the early steps of the sequence, that is, in occipital and superior temporal sulcus regions. The main abnormality was observed in mirror areas including the inferior frontal gyrus. Inferior frontal gyrus activations were spatially more scattered in ASD than control participants, and the signals were delayed and reduced in strength. Using fMRI, Martineau et al. (2010) found atypical activation in ASD during observation of human movement in various cerebral areas, including the motor cortex, the inferior frontal gyrus (pars opercularis), the parietal lobule, and the precuneus. There is also evidence that ASD adults exhibit structural abnormalities in cortical thickness in areas related to action observation (Hadjikhani et al., 2006; see Figure 2).

Other studies, however, fail to report functional abnormalities to action observation. Using EEG, Raymaekers et al. (2009) found significant mu wave suppression to self and observed movements in both high-functioning ASD children and typically developing children. Similarly, Fan et al. (2010) report that mu suppression over sensorimotor cortex when watching hand actions did not significantly differ in ASD and control participants. Oberman et al. (2008) found that mu suppression is sensitive to the degree of familiarity: in contrast to typically developing children, children with ASD show mu suppression but only when they can identify in some personal way with the observed movements (i.e., when observing their own movement or the movement of a familiar person).

## On and beyond the broken mirror hypothesis

Do individuals with ASD resonate to others' actions? The research discussed above identifies a number of effects that are preserved in ASD: individuals with ASD show compatibility effects to task-irrelevant action stimuli, demonstrate motor interference for simultaneous execution-observation of meaningless arm-movements, exhibited mu suppression when watching others actions (but see Oberman et al., 2005). Other features of the visuomotor resonant response, however, appear to be absent or abnormal. Below we consider some of the factors that modulate visuomotor resonance in neurotypical individuals and that, in our opinion, can be of help in interpreting apparently divergent results in ASD.

### Motor hierarchy

In accordance with the idea that the motor system is hierarchically organized (Grafton and Hamilton, 2007), motor resonance has been proposed to operate at different levels (Blakemore and Frith, 2005). At the lowest level there is resonance to movements as long as these are made (or believed to be made, see Stanley et al., 2007) by biological entities. At a higher level there is resonance to specific goal-directed actions. At an even higher level, motor resonance may be caused by intentions. Observer with ASD may resonate to others' action at some levels (goal, e.g., Bird et al., 2007), but not at other levels (intention; Pierno et al., 2006; Becchio et al., 2007).

### Biological tuning

A number of studies have demonstrated that, in neurotypical observers, human movements produce larger visuomotor resonance than artificial, impossible, or robotic movements (Castiello et al., 2002; Tai et al., 2004; Longo et al., 2008; Longo and Bertenthal, 2009; Liepelt and Brass, 2010). This been proposed to reflect tuning to both the form and kinematic profile of human movements (Press, 2011). Apparently divergent results in ASD may be interpreted assuming that observers with ASD are sensitive to the form (Bird et al., 2007), but not by the kinematics of the human movement (Pierno et al., 2006; Becchio et al., 2007). The finding of robotic tuning (Pierno et al., 2008) raise the interesting possibilities that observers with ASD might be more responsive to robotic than human kinematics.

### Input/output modulation

Visuomotor resonance is modulated by changes in spatial attention and feature selection (input modulation), as well as by social cognitive processes influencing the extent to which motor activation is inhibited or allowed to influence overt behavioral performance (output modulation; for review, see Heyes, 2011). Differences in the way observers with ASD distribute their attentional resources and process social stimuli may help to explain differences in motor resonance to different features of actions. For example, differences in output modulation by emotional cues have been observed for imitative movements (Grecucci et al., in press). Whereas typically developing controls showed a strong modulation (i.e., faster responses) of imitative responses when primed by social/emotional cues, children with ASD did not. The finding that gaze does not modulate motor facilitation and interference effects in ASD (Pierno et al., 2006; Becchio et al., 2007; Schilbach et al., 2011) adds to this view, suggesting that inability to read motor intention from gaze direction might contribute to abnormalities in the way visual information from observed actions is converted into a program for motor execution. It remains unclear whether the degree of familiarity is critical for the visuomotor resonant response to occur (Oberman et al., 2008).

### Heterogeneity of the ASD population

We know little about whether and how visuomotor resonance varies across the different diagnostic subcategories of ASDs. Some aspects of motor functioning and motion perception appear to vary across different clinical subpopulations within the autism spectrum (for review, see Kaiser and Shiffrar, 2009; Bhat et al., 2011). For example, there is evidence that, during the perception of locally oriented patterns, observers with high functioning autism show elevated motion coherence thresholds relative to typical observers, whereas observers with Asperger Syndrome do not (Spencer and O'Brien, 2006; Tsermentseli et al., 2008). Moreover, although both children with ASD and typically developing children show decreasing motion coherence thresholds with increasing age, this trend appears to be more pronounced for observers with ASD (Kaiser and Shiffrar, 2009). Diagnostic heterogeneity between subjects as well as age related differences might thus contribute to some of the variability of results across studies investigating visuomotor resonance.

## Visuomotor resonance in the context of social interaction

To date, researchers have specifically emphasized the potential contribution of visuomotor abnormalities to deficits in social cognition associated with autism. Dysfunction of visuomotor resonance mechanisms early in development may give rise to deficits in imitation (Iacoboni and Dapretto, 2006). It may underlie deficits in intention recognition (Cattaneo et al., 2007) and empathy (Gallese, 2006). Finally, by disrupting embodied simulation, dysfunction of visuomotor resonance may contribute to deficits related to more sophisticated mental abilities such as theory of mind and language (Oberman and Ramachandran, 2007). All these potential functions consider the role of visuomotor integration in social and communicative deficits in ASD. However, to the extent that the visuomotor resonance is forged by experience, abnormalities in visuomotor resonance may not only contribute to, but also depend on the difficulties that individuals with ASD encounter during social interaction.

This hypothesis is supported by recent findings suggesting that sensorimotor experience can enhance (Press et al., 2007), abolish (Heyes et al., 2005), and even reverse (Catmur et al., 2007, 2008) visuomotor resonance in human subjects. For example, training to perform one action (e.g., little finger movement) while observing another action (index finger movement) can reverse TMS-induced muscle-specific activation, so that after training the observation of index finger movement produces more activity in little finger than in index finger muscles (Catmur et al., 2007). In line with associative sequence learning models, this suggests that visuomotor resonance can be readily transformed by sensorimotor experience (Heyes, 2010).

Because in naturalistic settings much of sensorimotor experience is obtained through interaction with others, experiences that differ from those typically encountered during life may reconfigure the sensory-motor integration and change the way it operates. In this view, social deficits in ASD may play a constitutive role in abnormalities in visuomotor resonance. If abnormalities exist in the way individuals with ASD connect observed and executed movements, this might be, at least in part, because deficits in social interaction hinder sensorimotor learning.

### Conflict of interest statement

The authors declare that the research was conducted in the absence of any commercial or financial relationships that could be construed as a potential conflict of interest.
